# Gallic acid chemoprevention of oral carcinogenesis is associated with HSD11β2 upregulation and immune remodeling

**DOI:** 10.1038/s41598-026-50700-1

**Published:** 2026-04-27

**Authors:** Puja Upadhaya, Felipe F. Lamenza, Ravi Ramalingam, Kishan Kumar Nyati, Sushmitha Jagadeesha, Suvekshya Shrestha, Reegan Kehres, Natalie Kazmierowicz, Sonali Dasari, Shaheer Masood, Peyton Roth, Hasan Pracha, Steve Oghumu

**Affiliations:** Department of Pathology, Pelotonia Institute for Immuno-Oncology, The Ohio State University Comprehensive Cancer Center, College of Medicine, The Ohio State University Wexner Medical Center, 242A Evans Hall, 520 King Ave., Columbus, OH 43201 USA

**Keywords:** Gallic acid, Head and neck squamous cell carcinoma, Chemoprevention, 11β-hydroxysteroid dehydrogenase type 2, Glucocorticoids, Immune modulation, 4-nitroquinoline-1-oxide, Cancer, Cell biology, Oncology

## Abstract

Naturally derived phytochemicals such as gallic acid (GA) exhibit multitargeted anticancer properties and favorable safety profiles, yet the molecular mechanisms underlying their chemopreventive effects in head and neck squamous cell carcinoma (HNSCC) remain incompletely defined. Using a 4-nitroquinoline-1-oxide (4NQO)-induced oral carcinogenesis mouse model, we evaluated GA’s effects on tumor progression, immune modulation, and stress-hormone-related pathways. Phenotypic outcomes were assessed by histopathology, proliferation markers, and in vitro cytotoxicity assays. Transcriptomic changes were profiled by RNA sequencing, pathway enrichment and validation using RT-qPCR, Western blotting, ELISA, and flow cytometry. GA selectively inhibited proliferation of HNSCC cell lines (CAL27, SCC83) while sparing normal oral epithelial cells (TE1177). In vivo, GA reduced tumor burden and histopathologic severity without affecting body weight. RNA-seq analysis revealed coordinated modulation of immune and stress-response pathways, including upregulation of *Carmil2*, *Cd27*, and *Cd209d* and downregulation of *Fos*, *Pappa*, and *Hif1a*. GA increased HSD11β2 expression in vitro and in vivo and reduced cortisol in cAMP-stimulated HNSCC cells, findings consistent with reduced local glucocorticoid signaling. GA also increased IL-2 and decreased IL-10 in T cells, reduced monocytic MDSCs, and lowered PD-L1 on pro-inflammatory macrophages. Together, these findings identify HSD11β2/glucocorticoid metabolism as a potential axis associated with GA-mediated oral cancer chemoprevention.

**Supplementary Information:**

The online version contains supplementary material available at 10.1038/s41598-026-50700-1.

## Introduction

Cancer remains one of the foremost causes of morbidity and mortality worldwide, accounting for an estimated 9.6 million deaths annually^1^. Despite significant progress in understanding carcinogenesis and developing targeted therapies, the clinical effectiveness of conventional chemotherapeutic agents is often limited, primarily due to the emergence of chemoresistance and systemic toxicity^2,3^. To overcome these limitations, conventional agents are frequently administered at high doses to achieve therapeutic efficacy. However, this approach not only increases the risk of severe adverse effects but also activates compensatory survival mechanisms within tumor cells, ultimately leading to treatment failure^4,5^. It is well established that therapies targeting only one or a few pathways are insufficient for long-term cancer control, given the multifactorial nature of tumor progression and resistance^5,6^. As a result, recent research has increasingly focused on the development of multitargeted treatment strategies.

Naturally derived phytochemicals, plant-based compounds with broad spectrum biological activity have emerged as promising candidates in this domain. These agents can modulate multiple oncogenic and survival pathways simultaneously, often with reduced toxicity compared to synthetic drugs. Their chemosensitizing properties make them attractive for use in combination therapies aimed at enhancing the efficacy of standard treatments while minimizing adverse effects^7^. Among the malignancies actively being explored for phytochemical based interventions is head and neck squamous cell carcinoma (HNSCC), which arises from the mucosal epithelium of the oral cavity, pharynx, and larynx and represents one of the most common cancers in the head and neck region^8^. Despite advances in surgical resection, chemoradiotherapy, and immune checkpoint blockade with targeted therapies, the global 5-year survival rate for HNSCC remains at approximately 50–60%^8–11^. This limited prognosis is largely attributed to the biological and molecular heterogeneity of HNSCC, which is driven by diverse etiological factors, including tobacco use, alcohol consumption, and infection with high-risk human papillomavirus (HPV) strains^12–15^. Given these challenges, there is an urgent need to explore novel therapeutic strategies that are both effective and well tolerated.

Gallic acid (3,4,5-trihydroxybenzoic acid; GA) is a naturally occurring plant-derived polyphenol that has gained significant attention for its wide range of pharmacological properties, including its potential role in cancer prevention and therapy. GA is found abundantly in dietary sources such as berries, tea, and grapes^16–18^. Among dietary sources, black raspberries (BRBs; *Rubus occidentalis*) are particularly rich in ellagitannins, a class of hydrolyzable polyphenols that release both ellagic acid and GA during digestion^19^. BRBs have been reported to contain approximately 965.6 ± 2.9 mg of GA equivalents per gram, a significantly higher concentration compared to red raspberries (239.6–372.0 mg/100 g dry weight) and blackberries (226.0 mg/100 g)^19^. Previous studies by our group and others have demonstrated the high potential of BRBs and their phytochemicals as chemopreventive agents against a variety of cancers and inflammatory conditions^20–25^. These studies have shown that BRB administration not only reduces tumor burden in HNSCC but also modulates immune responses, alters metabolic and hormonal pathways, affects the expression of genes involved in oxidative stress and cancer progression, and attenuate glucocorticoid-mediated pro-tumorigenic signaling^21–25^. However, the specific bioactive compounds within BRBs responsible for these effects remain largely unidentified.

Over the years, GA has been reported to exhibit multiple biological activities, including potent pro-apoptotic^26^, anti-inflammatory^27^, and anticancer effects^28,29^. Supporting its role in cancer chemoprevention, in vivo studies have demonstrated the protective effects of GA against chemically induced carcinogenesis like colon cancer^30^ and prostate cancer^26,31^. GA not only has chemopreventive effects but also enhances immune checkpoint blockades by promoting T-helper-1-like Treg cells. In colorectal cancer, combining GA with anti-PD-1 reduces Treg function, downregulates Foxp3, boosts CD8^+^ T cell IFN-γ production, and limits tumor growth^32^. A recent study by Guimarães et al. (2023) provided evidence of GA’s protective effect in a 4-nitroquinoline 1-oxide (4NQO)-induced oral carcinogenesis model^33^. The study showed that GA treatment significantly reduced macroscopic tumor burden, decreased the incidence of oral squamous cell carcinoma (OSCC), and improved survival outcomes in mice exposed to 4NQO. Moreover, GA statistically lowered the histopathological grade of 4NQO induced lesions. However, the precise molecular mechanisms underlying these protective effects remain largely unexplored.

Given the chemopreventive potential of GA and the limited mechanistic understanding provided by existing in vivo studies, this work aims to elucidate the molecular pathways modulated by GA in oral carcinogenesis. Utilizing the well-established 4NQO mouse model of HNSCC, which closely mimics early events in human oral cancer development, we investigated the impact of GA on tumor progression, immune regulation, and local glucocorticoid metabolism. We further explored associated molecular mechanisms using transcriptomic profiling of tongue tissues from 4NQO induced GA treated and vehicle control mice. Understanding these pathways has the potential to reveal novel dietary-based strategies for cancer prevention and inform multitargeted approaches to overcome treatment resistance and improve patient outcomes.

## Methods

### Animals

Five-week-old C57BL/6 mice were purchased from The Jackson Laboratory and housed under standard laboratory conditions with a 12-h light/dark cycle, and free access to food and water. All animal procedures were conducted in accordance with the guidelines set by University Laboratory Animal Resources. At terminal sacrifice, mice were euthanized with compressed CO₂ using a regulator and flow meter, delivered into an uncharged chamber at 30–70% volume displacement per minute. CO₂ flow was maintained for ≥ 1 min after respiratory arrest, followed by cervical dislocation. The study protocol received approval from The Ohio State University Institutional Animal Care and Use Committee (Protocol #2018A00000054) and the Institutional Biosafety Committee.

### In vitro cell viability assay

Authenticated human oral cancer cell lines CAL27, SCC83 and the normal tongue epithelial cell line TE1177 were kindly provided by Dr. Weghorst. Cell viability was assessed using the MTT assay kit (ab211091). Cells were seeded in 96-well plates at a density of 5 × 10^3^ cells per well in DMEM supplemented with 10% FBS. After 24 h, cells were treated with GA at various concentrations (0, 12.5, 25, 50, 100, 200, 400, and 800 μM). After 24 h of treatment, the culture medium was replaced with 50 μl of serum-free medium and 50 μl of MTT solution (0.5 mg/ml). After a 3-h incubation at 37 °C, formazan crystals were dissolved in 150 μl MTT solvent, and absorbance was measured at 590 nm.

### HNSCC model

To induce oral carcinogenesis, mice were administered 4-nitroquinoline 1-oxide (4NQO; Sigma-Aldrich, St. Louis, MO, USA; Cat# N8141) in their drinking water at a concentration of 100 µg/mL. The solution was freshly prepared, protected from light in foil-wrapped bottles, and replaced every week. 4NQO exposure continued for 16 weeks, after which mice were provided regular drinking water until the time of terminal sacrifice. A total of 23 mice were used in this study, including 11 mice in the vehicle group and 12 mice in the GA-treated group.

Four weeks after the initiation of 4NQO exposure, mice were randomly assigned to receive either GA or vehicle control. GA (Sigma Aldrich, MO, USA; Cat# G7384) was prepared as a stock solution in DMSO, then diluted in a vehicle consisting of 0.5% methyl cellulose and 0.025% Tween-20. Mice in the treatment group received GA at 100 mg/kg body weight via oral gavage five times per week, while control animals received an equal volume of vehicle using the same schedule. Treatment continued until the day of terminal sacrifice.

Tongues of mice were collected at the end of the 4NQO experiment and examined for tumor formation. Tumor size and number were recorded. Macroscopic tumors were identified by visual inspection and photographed with a ruler included in the image for scale reference. Tumor area was quantified using ImageJ software based on the captured images. Only lesions visible to the naked eye were considered for macroscopic tumor quantification. Lesions that were not clearly visible macroscopically were further evaluated histologically using hematoxylin and eosin (H&E) staining to allow classification and characterization of benign and malignant lesions. Histopathological evaluation was used as the standard method for lesion classification. Due to the anatomical location and progression characteristics of lesions in the 4NQO oral carcinogenesis model, longitudinal measurements of tumor burden during the experimental period are generally not feasible; therefore, tumor burden was assessed at the study endpoint.

### Flow cytometry

Single-cell suspensions from lymph nodes and spleens were stained with fluorochrome-conjugated antibodies against CD3 (Cat#100216), CD4 (Cat#100414), CD8 (Cat#100734), PD-1 (Cat#135218), CTLA-4 (Cat#106310), TIGIT (Cat#156104), LAG-3 (Cat#125219), Ly6C (Cat#128041), Ly6G (Cat#127608), CD11b (Cat#101206), CD206 (Cat#141716), F4/80 (Cat#123132), and PD-L1 (Cat#124324), all from BioLegend (San Jose, CA, USA). For intracellular staining, cells were stimulated with PMA/ionomycin (BioLegend) for 6 h, followed by staining with granzyme B (Cat#372208), IFN-γ (Cat#505822), IL-2 (Cat#503833), and IL-10 (Cat#505034) antibodies. Data were acquired on a FACS Celesta flow cytometer (BD Biosciences, San Jose, CA), and analyzed using FlowJo software (FlowJo, Ashland, OR).

### Histology

Tongue tissues were fixed in formalin, embedded in paraffin, and stored in 70% ethanol following established protocols^24^. Tissue sections (5 µm) were stained with hematoxylin and eosin (H&E). For immunohistochemistry (IHC), staining was performed as previously described^34^ using antibodies against Ki-67 (Invitrogen, MA5-14520) and HSD11β2 (Proteintech, 14192–1-AP). The following day, a biotinylated goat anti-rabbit secondary antibody (Vector Labs, Newark, CA, USA; BA-1000) was applied. Areas with positive staining were quantified using ImageJ.

### ELISA

Competitive ELISA (R&D Systems) were performed to quantify cortisol (KGE008B) and corticosterone (KGE009) in culture supernatants and serum. Absorbance was measured at 450 nm and 540 nm using a SpectraMax 190 microplate reader (Molecular Devices, San Jose, California, USA).

### Western blot

Total protein was extracted from CAL27 and SCC83 cells from control and treatment groups using RIPA buffer supplemented with protease inhibitors. Twenty micrograms of protein were separated on a 10% SDS‑PAGE gel and transferred to PVDF membranes. Membranes were blocked with 5% milk for 1 h, then incubated with anti‑HSD11β2 (Proteintech, 14192–1-AP). HRP‑conjugated anti‑rabbit secondary antibody (Thermo Fisher Scientific, 31460) was applied, and detection was performed using ECL substrate. Band intensities were quantified using ImageJ.

For immunoblot analysis, membranes were cut prior to antibody incubation to allow separate probing of the target protein (HSD11β2) and the housekeeping protein (GAPDH) for each cell lysate (CAL27 and SCC83), thereby avoiding repeated stripping and reprobing of the same membrane. Blot images presented in the main figures were cropped for clarity to highlight the relevant bands. Uncropped images of the corresponding blot sections, including membrane edges where available, as well as images from all experimental replicates and additional exposure images, are provided in the Supplementary Figs. 1A-D and 2A-D.

### RT-qPCR

RNA was extracted with TRIzol, and cDNA was synthesized using the High‑Capacity cDNA Reverse Transcription Kit (Applied Biosystems). RT‑qPCR was conducted using SYBR Green Master Mix (Thermo Fisher) on a Bio‑Rad CFX384. Primers for HSD11β2 were designed using PrimerBank (https://pga.mgh.harvard.edu/primerbank/).

### RNA sequencing

Tongue samples from GA-treated and vehicle-treated 4NQO mice were processed for bulk RNA-seq. RNA quality was assessed by FastQC (v0.11.7). Reads were aligned to the *Mus musculus* reference genome (mm10) using HISAT2. Uniquely mapped reads were quantified at the gene level with featureCounts (Subread). Lowly expressed genes were filtered prior to normalization. Differential expression analysis was performed in edgeR (TMM normalization; quasi-likelihood framework)^35^, and genes with FDR < 0.1 and |log₂ fold change|≥ 0.58 were considered significant^36^. Gene Ontology enrichment was performed with clusterProfiler^37^.

### Statistics

For non‑omics experiments, data are presented as mean ± SE. Two‑tailed unpaired Student’s t‑tests were used for two‑group comparisons; Welch’s correction was applied when variances were unequal. Non‑parametric Mann–Whitney tests were used when normality assumptions were not met. A threshold of *p* < 0.05 was considered significant. For RNA‑seq, multiple‑testing correction used Benjamini–Hochberg FDR with a significance threshold of FDR < 0.1. Analyses were performed using GraphPad Prism (v8.0.2) and R (v4.0.2).

## Results

### Gallic acid inhibits HNSCC proliferation and reduces tumor burden

**Fig. 1:**
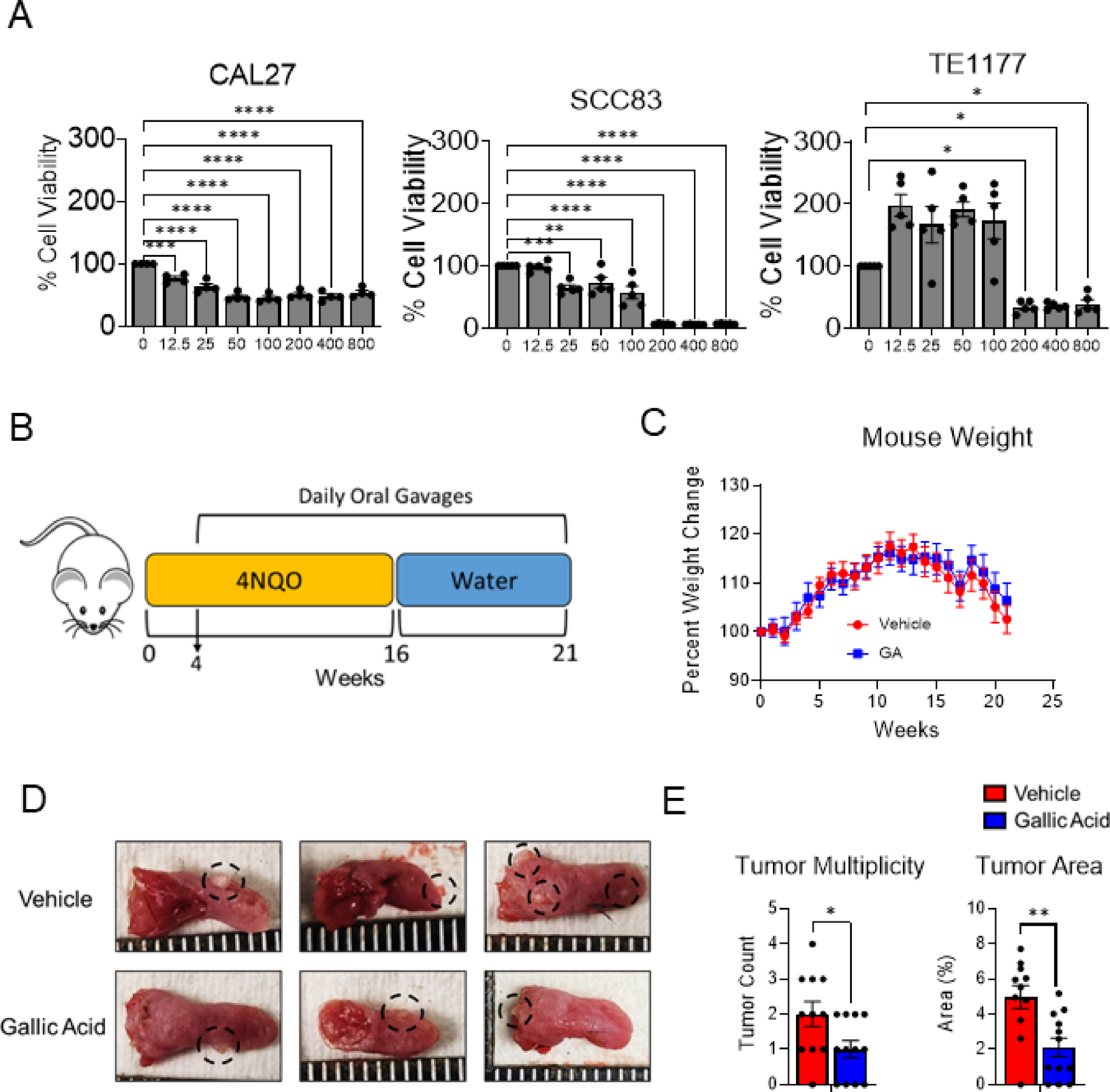
Gallic acid reduces HNSCC tumor burden in vitro and in vivo. (**A**) Bar graph showing the percentage of cell proliferation in CAL27, SCC83, and TE1177 cell lines treated with increasing concentrations of GA (0, 12.5, 25, 50, 100, 200, 400, and 800 µM), as measured by MTT assay. (**B**) Schematic representation of the 4NQO-induced oral carcinogenesis mouse model, with GA administration (100 mg/kg) beginning at week 4 of 4NQO exposure. (**C**) Weekly measurement of percent body weight change in mice treated with vehicle or GA throughout the study duration. (**D**) Representative images of excised tongues from each group at endpoint; tumor regions are circled with black dotted lines. (**E**) Quantification of tumor multiplicity and tumor area at terminal sacrifice in mice treated with vehicle or GA. Data are presented as mean ± SE * *P* < 0.05; ** *P* < 0.01; *** *P* < 0.001; and **** *P* < 0.0001.

Given GA’s reported anticancer properties, we first assessed its cytotoxicity against oral cancer cells and its selectivity for malignant versus normal epithelial cells. In vitro viability assays showed that GA significantly reduced viability of CAL27 and SCC83 oral cancer cell lines in a dose-dependent manner at concentrations as low as 12.5–100 μM, while TE1177 normal oral epithelial cells exhibited minimal cytotoxicity under the same conditions (Fig. 1A).

To determine whether these effects translate in vivo, we utilized the 4NQO-induced oral carcinogenesis mouse model (Fig. 1B), which closely mimics early events in human oral cancer development. GA was administered at 100 mg/kg via oral gavage five times per week. Tumor development was observed in 10 out of 11 mice (91%) in the vehicle group and 8 out of 12 mice (67%) in the GA-treated group. GA-treated mice displayed a marked reduction in tongue tumor multiplicity and tumor area compared to vehicle controls (Fig. 1D-E). Importantly, GA administration did not affect body weight throughout the study, indicating good tolerability (Fig. 1C).

### Gallic acid reduces histopathological severity and cellular proliferation

**Fig. 2:**
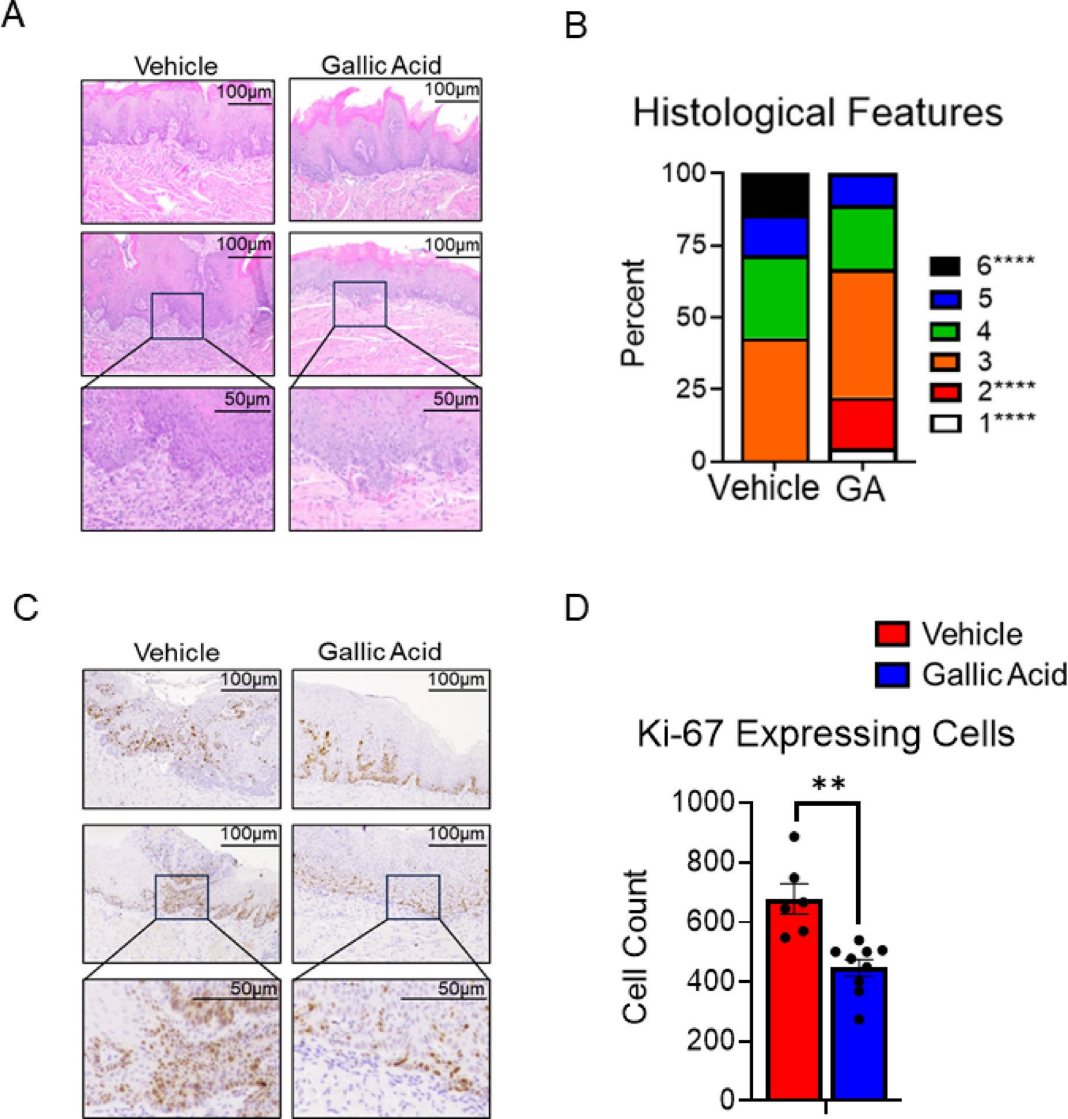
Gallic acid reduces histopathological severity and cellular proliferation in 4NQO-induced oral lesions. (**A**) Representative H&E-stained sections of tongue tissues from mice treated with either vehicle or GA, shown at 100 × and 200 × total magnification. (**B**) Bar graphs show the distribution (%) of the most histologically advanced lesions in each group. Lesions were classified as hyperplasia (1), mild dysplasia (2), moderate dysplasia (3), high-grade dysplasia (4), carcinoma in situ (5), or squamous cell carcinoma (6) in mice treated with vehicle or GA. (**C**–**D**) Representative images and quantitative analysis of Ki-67 immunohistochemical staining in tongue tissues from vehicle and GA treated mice at 100 × and 200 × magnification. Positive staining was assessed from at least five randomly selected fields per sample. Data are presented as mean ± SE. ** P < 0.01; ****P < 0.0001.

To evaluate whether GA alters the progression of oral lesions at the histological level, we examined tongue tissues for lesion severity and proliferation markers. Histological analysis revealed that GA-treated mice predominantly exhibited hyperplasia and mild dysplasia, whereas vehicle-treated mice developed invasive squamous cell carcinoma (Fig. 2A–B). Immunohistochemical staining for Ki-67 demonstrated significantly lower proliferation marker expression in GA-treated lesions compared to vehicle treated controls (Fig. 2C–D), supporting GA’s role in suppressing tumor cell proliferation.

### Gallic acid induces distinct transcriptional changes in immune regulation and stress response

**Fig. 3:**
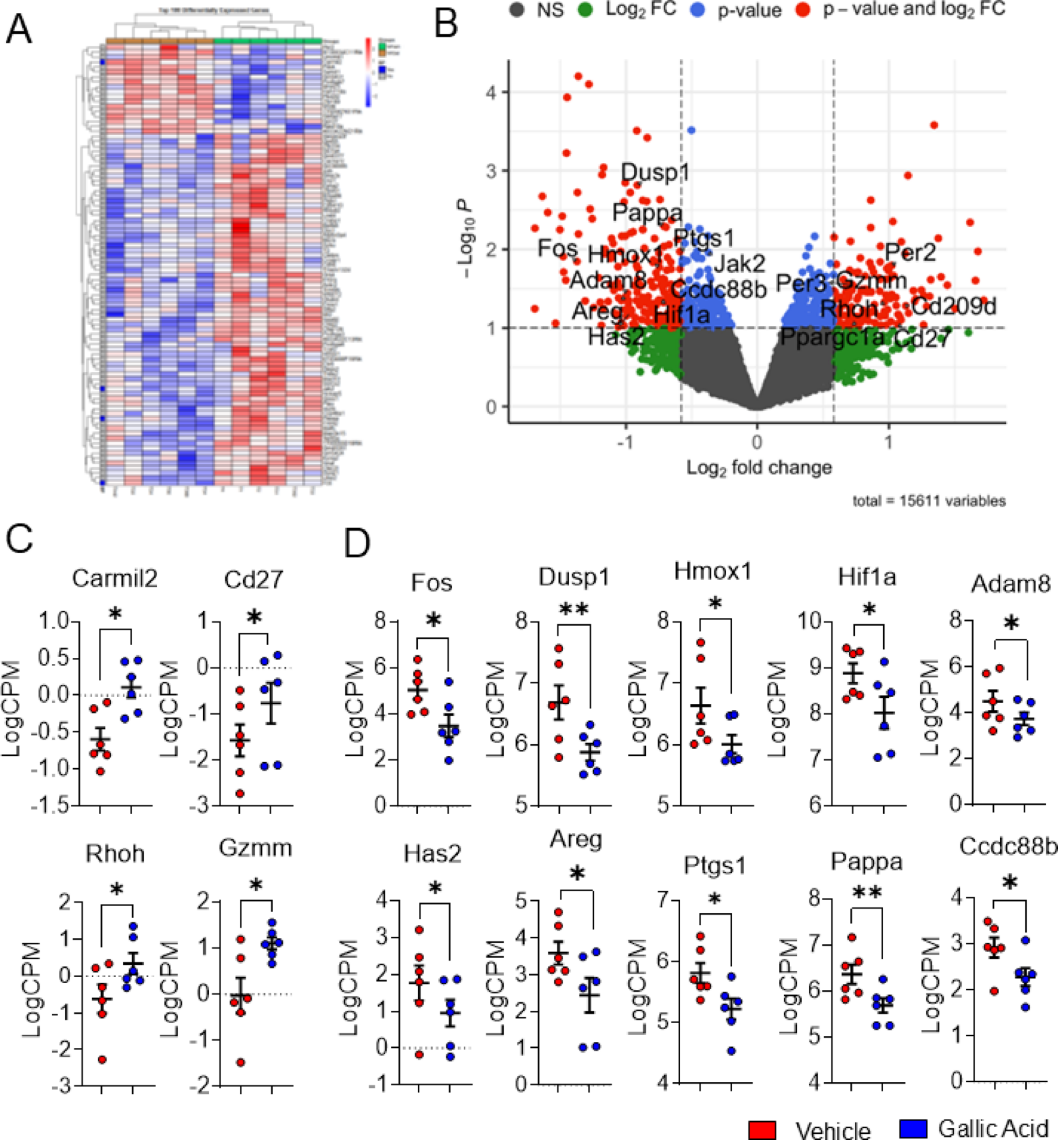
Gallic acid induces differential regulation of immunoregulatory genes in mouse tongue tissue. (**A**) Hierarchical clustering and heatmap of the top 100 significantly differentially expressed genes between GA treated and vehicle treated mice. The color scale represents the log₂ fold-change values, with red indicating upregulation and blue for downregulation. (**B**) Volcano plot illustrates several immune-associated genes prominently highlighting differentially expressed genes. (**C**–**D**) Scatterplots of selected genes affected by GA treatment. Genes associated with immune regulation are shown, with *Carmil2*, *Cd27*, *Gzmm* and *Rhoh* markedly upregulated, whereas *Fos*, *Dusp1*, *Hmox1*, *Hifa*, *Adam8*, *Has2*, *Areg*, *Ptgs1*, *Pappa*, and *Ccdc88b* are significantly downregulated in the GA treatment group.

To uncover molecular pathways underlying GA’s chemopreventive effects against HNSCC, we performed RNA sequencing on tongue tissues from GA-treated and control mice. Hierarchical clustering and volcano plots revealed distinct gene expression profiles between groups (Fig. 3A-B). Differential expression analysis identified significantly modulated genes (log₂ fold change ≥ 0.58, FDR < 0.1), which clustered into immune regulation, stress response, and metabolic adaptation pathways. RNA-seq revealed coordinated changes in immune related and stress response programs following GA treatment. Immune/costimulatory features included upregulation of *Carmil2*, *Cd27*, *Cd209d*, *Gzmm*, and *Rhoh* genes associated with T cell costimulation and immune effector activity (Fig. 3B-C). Conversely, multiple stress responsive and immediate early genes, including *Fos*, *Dusp1*, *Hmox1*, *Hif1a*, *Adam8*, *Areg*, *Has2*, and *Ptgs1*, were downregulated, along with additional remodeling associated genes such as *Pappa*, *Ccdc88b*, and *Jak2* (Fig. 3B and D). Circadian/metabolic regulators (*Per2*, *Per3*, *Ppargc1a*) were upregulated (Fig. 3B), suggesting a shift toward a less stress reactive transcriptional state. Collectively, these transcriptomic changes suggest that GA promotes immune activation while suppressing stress-induced pro-tumorigenic signaling, potentially contributing to its chemopreventive effects.

### Gallic acid upregulates HSD11β2 and reduces glucocorticoid activity

Given the observed changes in stress response pathways as revealed by our RNA seq data, we examined whether GA affects local glucocorticoid metabolism, a central regulator of cellular stress signaling. Because prereceptor control of glucocorticoid signaling is governed by 11β hydroxysteroid dehydrogenases, particularly HSD11β2 which converts active cortisol (corticosterone in mice) to inactive cortisone (11 dehydrocorticosterone), we evaluated its expression following GA treatment.

**Fig. 4:**
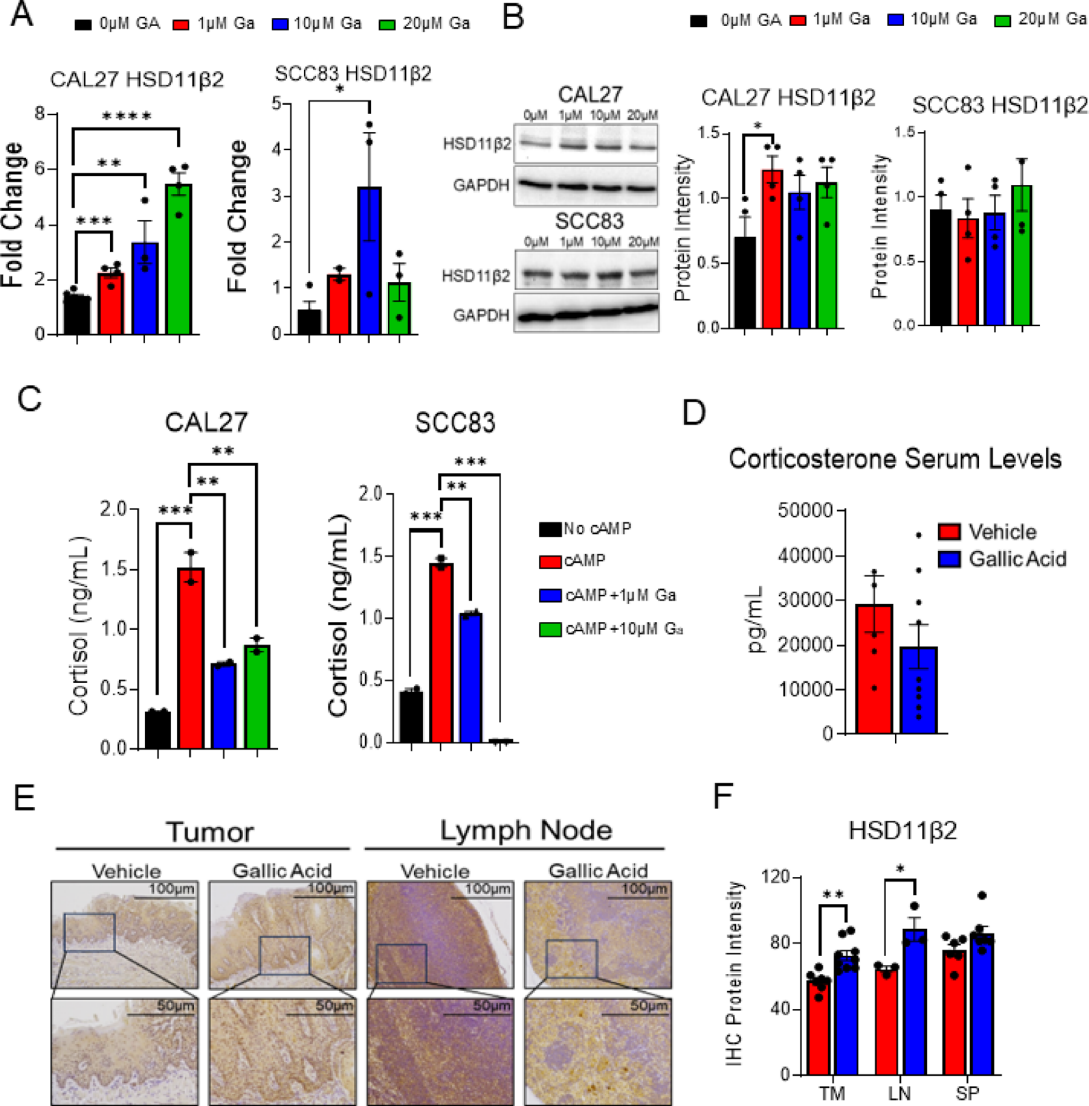
Gallic acid upregulates HSD11β2 expression in HNSCC in vitro and in vivo. (**A**) Gene expression levels of HSD11β2 in CAL27 and SCC83 HNSCC cell lines following treatment with increasing concentrations of GA (0, 1, 10, and 20 µM). (**B**) Representative Western blot images and corresponding densitometric analysis of HSD11β2 and GAPDH protein expression in CAL27 and SCC83 cells treated with 0, 1, 10, and 20 µM GA. Cropped blot images are shown for presentation clarity. Uncropped images of the corresponding membranes, including membrane edges where available, and images from all experimental replicates are provided in the Supplementary Figs. 1A-D and 2A-D. (**C**) Cortisol levels in the supernatants of CAL27 and SCC83 cells measured by ELISA following treatment with 0, 1, and 10 µM GA, with or without cAMP stimulation. (**D**) Serum corticosterone levels measured by ELISA in mice treated with vehicle or GA. (**E**–**F**) Representative immunohistochemistry images (100 × and 200 × magnification) showing HSD11β2 staining in tumors and lymph nodes from mice treated with vehicle or GA. Images were captured from at least five randomly selected fields per tissue. (**F**) Quantitative analysis of HSD11β2 IHC staining in tumors, lymph nodes, and spleens from both treatment groups. Data are presented as mean ± SE. *P < 0.05; **P < 0.01; ***P < 0.001; ****P < 0.0001.

In CAL27 and SCC83 cells, GA induced a dose dependent increase in HSD11β2 mRNA and protein expression (Fig. 4A-B, Supplementary Figs. 1 and 2). To functionally probe steroidogenic output under a defined stress like stimulus, we co-treated cells with cAMP, which augments steroidogenic signaling, and quantified cortisol in culture supernatants. GA reduced cortisol levels in cAMP stimulated conditions in both cell lines (Fig. 4C), consistent with enhanced inactivation of active glucocorticoids. Extending these observations in vivo, GA treated mice exhibited higher HSD11β2 immunoreactivity in tongue tumors and draining lymph nodes relative to vehicle controls, with a trend toward lower circulating corticosterone (Fig. 4D-F).

Together with the stress pathway transcriptional profile, these data indicate that GA not only reprograms stress response gene expression but also lowers effective glucocorticoid signaling capacity via HSD11β2 upregulation, providing a coherent link between transcriptomic evidence of attenuated stress signaling and biochemical readouts of reduced glucocorticoid activity.

### Gallic acid enhances T cell effector potential without driving immune exhaustion

**Fig. 5:**
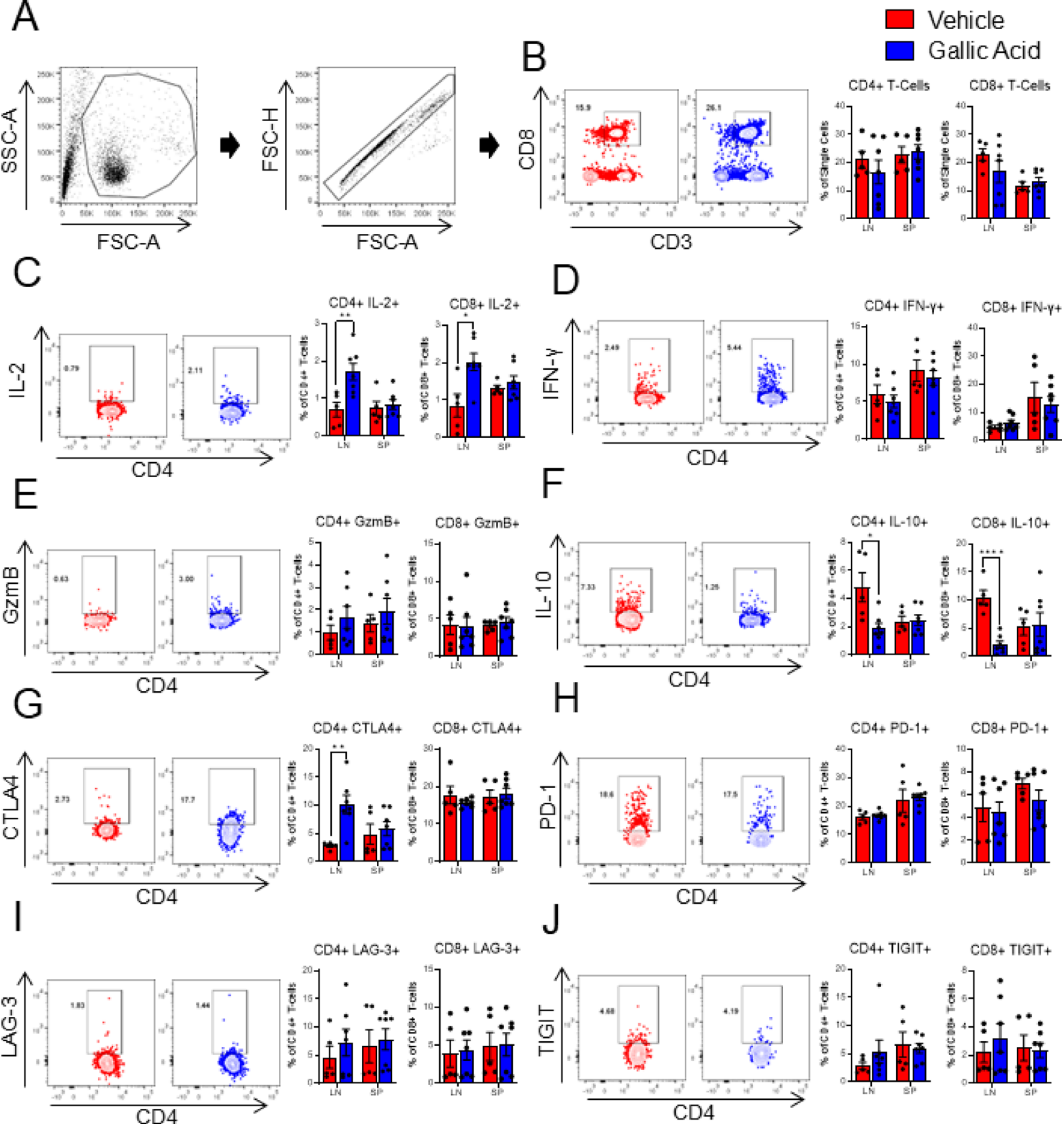
Gallic acid modulates T cell recruitment and co-inhibitory marker expression. (**A**) Representative flow cytometry plots and quantitative graphs depicting the percentages of various T cell subsets in the lymph nodes and spleens of mice treated with vehicle or GA. (**B**) Frequencies of CD3⁺CD4⁺ and CD3⁺CD8⁺ T cells. (**C**–**F**) Cytokine and effector molecule production, including IFN-γ (**D**), IL-2 (**C**), granzyme B (GzmB) (**E**), and IL-10 (**F**). (**G**–**J**) Expression of co-inhibitory receptors CTLA-4 (**G**), PD-1 (**H**), LAG-3 (**I**), and TIGIT (**J**). Data are presented as mean ± SE. *P < 0.05; **P < 0.01; ****P < 0.0001.

To determine whether GA modulates anti-tumor immunity during HNSCC, we analyzed T cell proliferation, effector function, and cytokine expression in secondary lymphoid organs. Flow cytometric analysis showed no significant differences in CD4⁺ or CD8⁺ T cell frequencies between GA-treated and control mice in the lymph nodes or spleen (Fig. 5A-B). However, functional profiling revealed increased IL-2 expression and reduced IL-10 production in lymph node T cells from GA-treated mice (Fig. 5C, F), consistent with enhanced effector priming. Granzyme B and IFN-γ levels in CD4^+^ and CD8^+^ T cells remained unchanged (Fig. 5D-E). Expression of immune checkpoint markers was largely stable, including PD-1, LAG-3 and TIGIT, except for a selective increase in CTLA-4 on CD4⁺ T cells in draining lymph nodes (Fig. 5G-J). These findings suggest that during HNSCC chemoprevention, GA enhances T cell effector potential without driving broad immune exhaustion.

### GA modulates myeloid cell subsets and PD-L1 expression

**Fig. 6:**
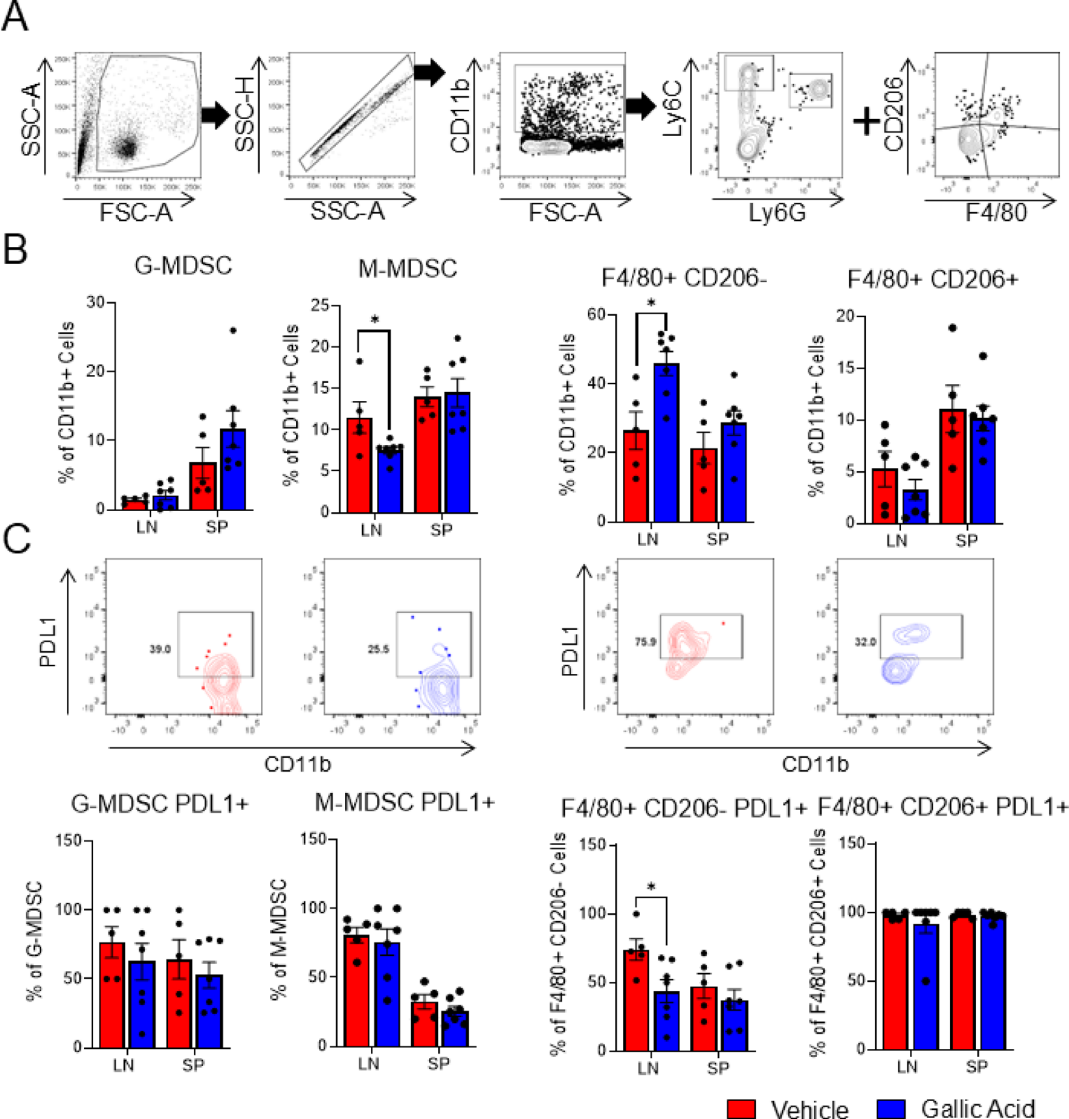
Myeloid cell population under gallic acid treatment. (**A**) Representative flow cytometric plots demonstrating gating strategy for G-MDSCs, M-MDSCs, F4/80 + CD206 + , and F4/80 + CD206- myeloid cell populations in the lymph nodes and spleens of cancer bearing mice. (**B**) Bar graphs depicting percentage of G-MDSCs, M-MDSCs, F4/80 + CD206-, and F4/80 + CD206 + myeloid cells in the lymph nodes and spleens of mice treated with vehicle or GA. (**C**–**D**) Representative flow plots and bar graphs of G-MDSCs, M-MDSCs, F4/80 + CD206 + , and F4/80 + CD206- myeloid cells expressing PD-L1 in the lymph nodes and spleens of mice treated with vehicle or GA. Data are presented as mean ± SE * *P* < 0.05.

To further characterize the immune microenvironment, we analyzed myeloid cell populations (Fig. 6A) and checkpoint ligand expression. GA treatment significantly reduced monocytic MDSCs (CD11b^+^Ly6G^-^Ly6C^hi^) in lymph nodes, while polymorphonuclear MDSCs (CD11b^+^Ly6G^+^Ly6C^lo^) remained unchanged (Fig. 6B). GA also increased pro-inflammatory macrophages (CD11b^+^F4/80⁺CD206⁻) without affecting M2-like macrophages (CD11b^+^F4/80⁺CD206^+^). Notably, PD-L1 expression was reduced on pro-inflammatory macrophages in tumor-draining lymph nodes (Fig. 6C-D). These findings are consistent with a model in which GA may reduce myeloid-driven immunosuppression during oral carcinogenesis.

## Discussion

This study demonstrates the chemopreventive potential of GA in HNSCC and provides mechanistic insights into the biological pathways associated with its activity. Using the 4NQO-induced oral carcinogenesis model, which closely recapitulates early events in human HNSCC^38^, we show that GA reduces tumor burden, suppresses histopathological progression, and enhances anti-tumor immunity without systemic toxicity. This work extends prior GA studies, which predominantly emphasized direct apoptotic and anti-inflammatory effects^26,28–31^, by identifying associations between GA treatment, immune remodeling, and HSD11β2/GC-related transcriptional shifts. It also integrates with earlier work showing that berry-derived phytochemicals modulate immune and hormonal axes in the oral mucosa^21–25^, and that GA may enhance responsiveness to immune checkpoint blockade in other tumor models^32^.

HNSCC remains a major clinical challenge due to tumor heterogeneity, chemoresistance, and treatment-related toxicity^8–11^. Conventional therapies often fail to achieve durable responses, underscoring the need for multitargeted strategies^5,6^. Phytochemicals such as GA offer broad-spectrum activity with favorable safety profiles^7,16–18^. Our findings confirm GA’s selective cytotoxicity toward oral cancer cells while sparing normal epithelial cells, consistent with prior studies in prostate and colon cancer models^26,30^. Importantly, GA’s ability to reduce tumor multiplicity and histological severity without affecting body weight supports its translational potential for long-term preventive use^39,40^.

Transcriptomic analysis showed upregulation of co-stimulatory and immune-associated genes (*Carmil2*, *Cd27*, *Cd209d*, *Gzmm*, *Rhoh*) and broad downregulation of immediate-early and stress-response mediators (*Fos*, *Dusp1*, *Hmox1*, *Hif1a*, *Adam8*, *Areg*, *Has2*, *Ptgs1*, *Pappa*, *Ccdc88b*, *Jak2*), a pattern consistent with a more immune-supportive and less stress-reactive transcriptional landscape during GA exposure. Carmil2, a scaffold protein essential for CD28-mediated NF-κB signaling, plays a critical role in T cell co-stimulation and effector function^41,42^. Its induction by GA suggests enhanced T cell activation and improved immune surveillance. Flow cytometric analysis corroborated these findings, showing increased IL-2 expression and reduced IL-10 production, which collectively favor a pro-inflammatory, anti-tumor immune environment^43^. Notably, GA did not broadly alter checkpoint markers such as PD-1, LAG-3, or TIGIT, indicating that immune activation occurs without driving T cell exhaustion. The selective increase in CTLA-4 on CD4⁺ T cells may represent a physiological feedback mechanism to fine-tune heightened immune activation^44,45^.

GA also reshaped the myeloid compartment by reducing monocytic MDSCs and decreasing PD-L1 expression on pro-inflammatory macrophages, changes that likely diminish immunosuppressive signaling within the tumor microenvironment^46^. These findings align with emerging evidence that targeting myeloid-derived suppressor cells and checkpoint ligand expression can restore anti-tumor immunity in HNSCC^34^.

A major mechanistic insight from this study is GA’s ability to upregulate HSD11β2, an enzyme that inactivates glucocorticoids by converting cortisol to cortisone^47,48^. Glucocorticoids are widely used in cancer therapy to manage treatment-related toxicities, yet they can promote tumor progression and immune suppression^49^. Our data suggest that GA attenuates glucocorticoid-driven oncogenic signaling by enhancing HSD11β2 expression and reducing active cortisol/corticosterone levels. This represents a previously underexplored axis of chemoprevention and may have clinical implications for patients receiving exogenous glucocorticoids during HNSCC treatment.

By simultaneously modulating immune responses and glucocorticoid metabolism, GA addresses two critical pathways that contribute to HNSCC progression. These multitargeted effects position GA as a promising candidate for dietary-based chemoprevention and as a potential adjunct to conventional therapies. Given its abundance in commonly consumed foods such as berries and tea^16–19^, GA offers a practical and safe approach for population-level cancer prevention strategies.

Despite these promising findings, several limitations warrant consideration. While transcriptomic and immune profiling provide mechanistic insights, causal relationships between specific gene changes and phenotypic outcomes remain to be validated using genetic or pharmacologic approaches. Future studies should employ pharmacologic manipulation of glucocorticoid pathways or genetic perturbation (CRISPR or siRNA) strategies to confirm the role of HSD11β2 in mediating GA’s effects and assess long-term safety and efficacy in combination with standard therapies, particularly under conditions of exogenous glucocorticoid administration. Additionally, clinical trials will be essential to determine optimal dosing and bioavailability in humans.

In summary, GA shows chemopreventive potential in 4NQO-induced experimental HNSCC model and may involve complementary mechanisms including selective cytotoxicity, enhancement of anti-tumor immunity, and modulation of glucocorticoid signaling via HSD11β2 upregulation. These findings warrant further investigation to determine the potential role of GA in oral cancer prevention and therapy.

## Supplementary Information

Below is the link to the electronic supplementary material.

Supplementary Material 1

## Data Availability

The datasets generated and analyzed during the current study are available in the Gene Expression Omnibus (GEO) repository, GSE318223.
