# Supplementary material for: Gallic acid chemoprevention of oral carcinogenesis is associated with HSD11β2 upregulation and immune remodeling
Source: Sci Rep. 2026 Apr 27;16:19285. doi: 10.1038/s41598-026-50700-1 (PMC13284332; doi:10.1038/s41598-026-50700-1)
Supplement: Supplementary file 1 — Supplementary Material 1 [file 41598_2026_50700_MOESM1_ESM.pptx]

## Slide 1
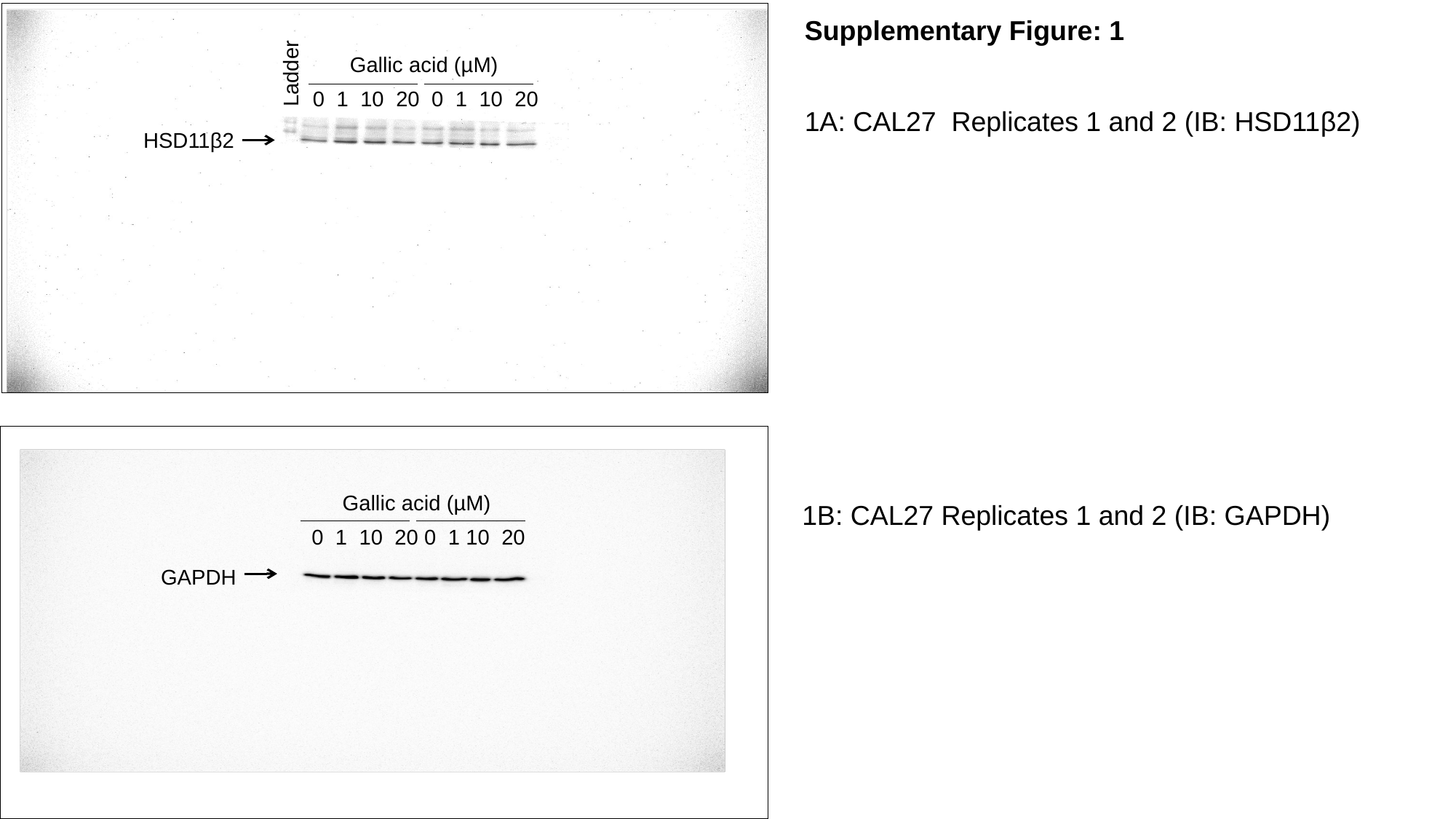

Supplementary Figure: 1
Gallic acid (µM)
0 1 10 20 0 1 10 20
HSD11β2
Ladder
1A: CAL27 Replicates 1 and 2 (IB: HSD11β2)
Gallic acid (µM)
0 1 10 20 0 1 10 20
1B: CAL27 Replicates 1 and 2 (IB: GAPDH)
GAPDH

## Slide 2
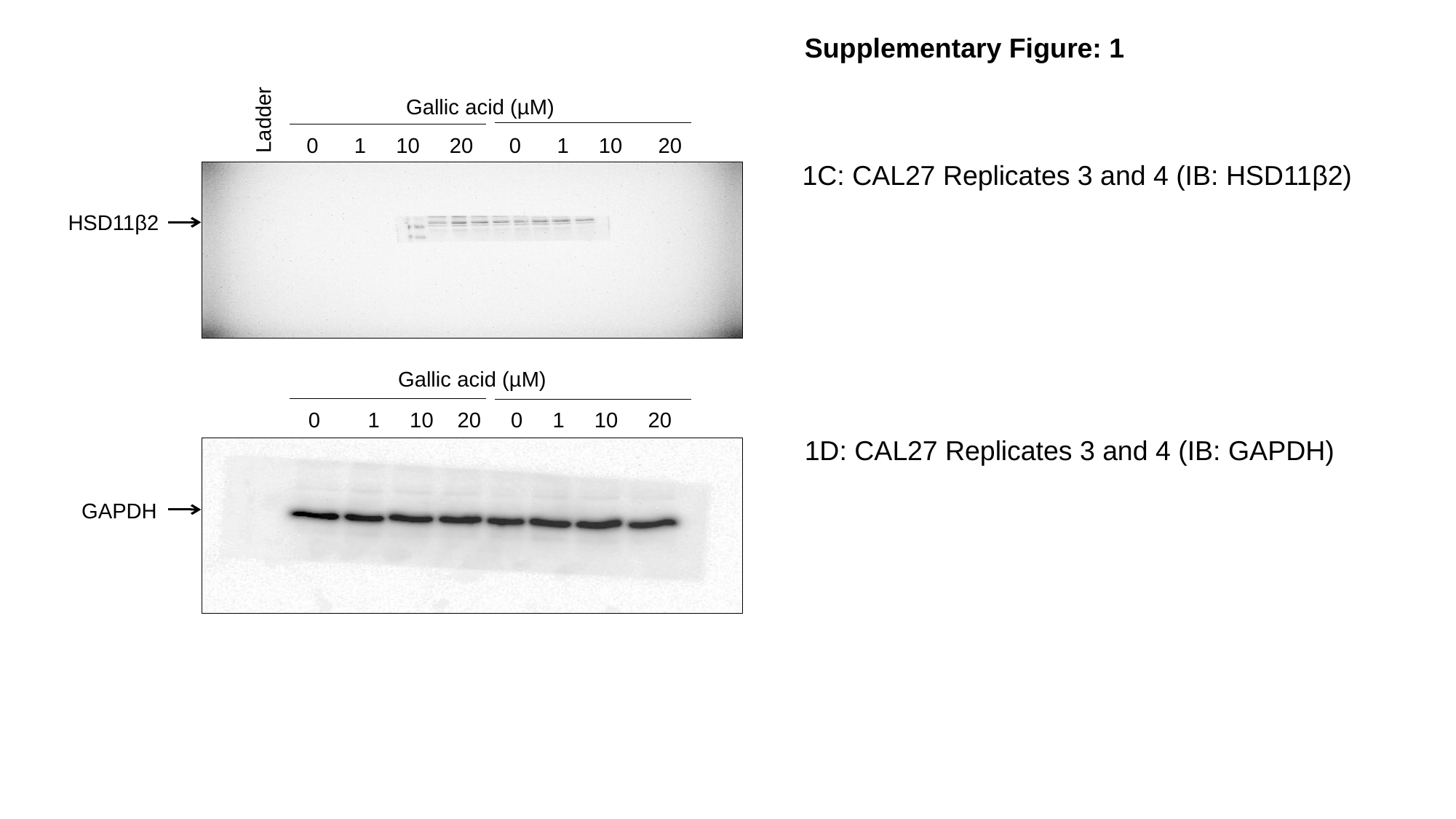

Supplementary Figure: 1
Gallic acid (µM)
 0 1 10 20 0 1 10 20
HSD11β2
Ladder
1C: CAL27 Replicates 3 and 4 (IB: HSD11β2)
Gallic acid (µM)
 0 1 10 20 0 1 10 20
GAPDH
1D: CAL27 Replicates 3 and 4 (IB: GAPDH)

## Slide 3
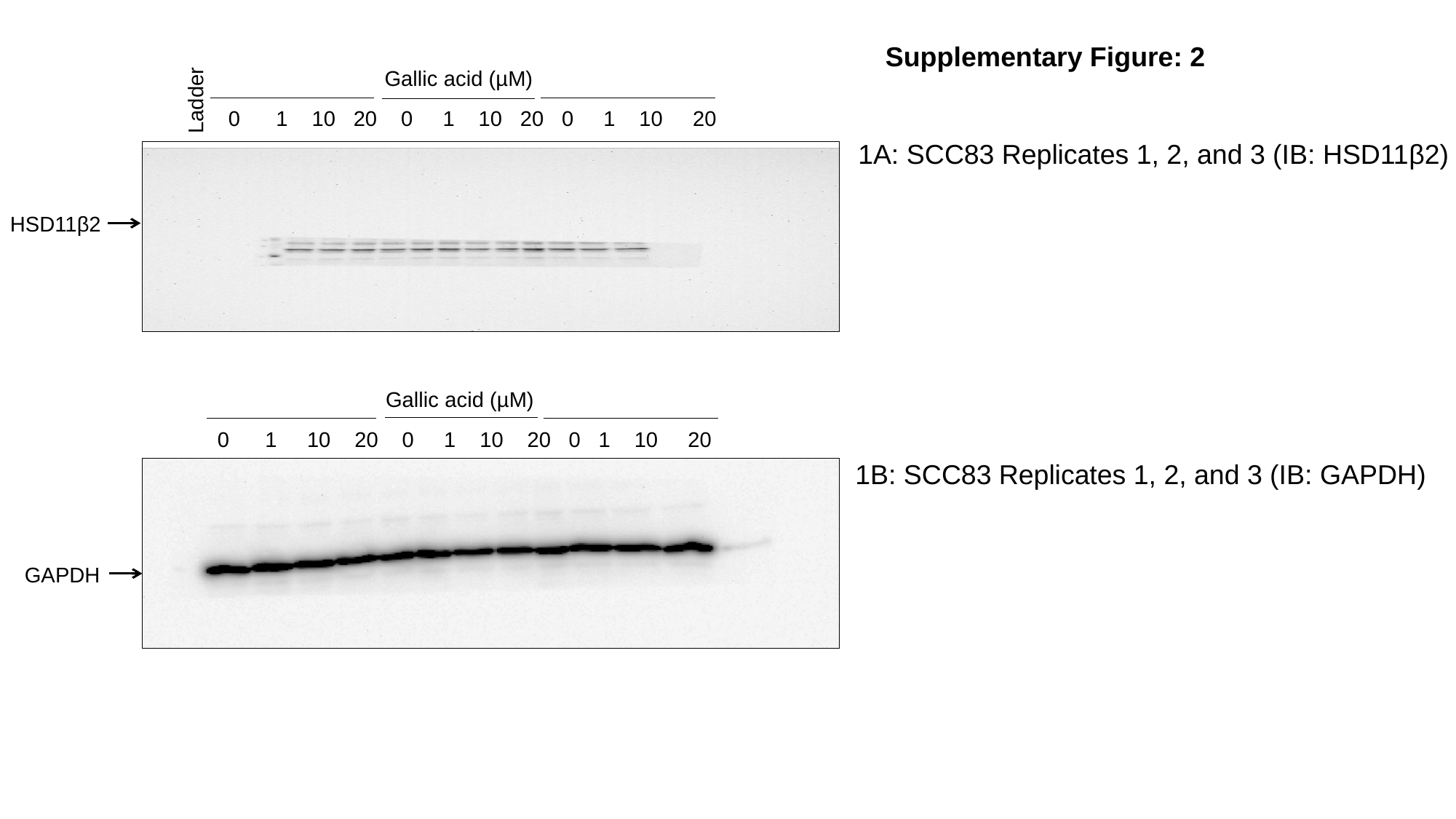

Supplementary Figure: 2
Gallic acid (µM)
 0 1 10 20 0 1 10 20 0 1 10 20
HSD11β2
Ladder
1A: SCC83 Replicates 1, 2, and 3 (IB: HSD11β2)
Gallic acid (µM)
0 1 10 20 0 1 10 20 0 1 10 20
GAPDH
1B: SCC83 Replicates 1, 2, and 3 (IB: GAPDH)

## Slide 4
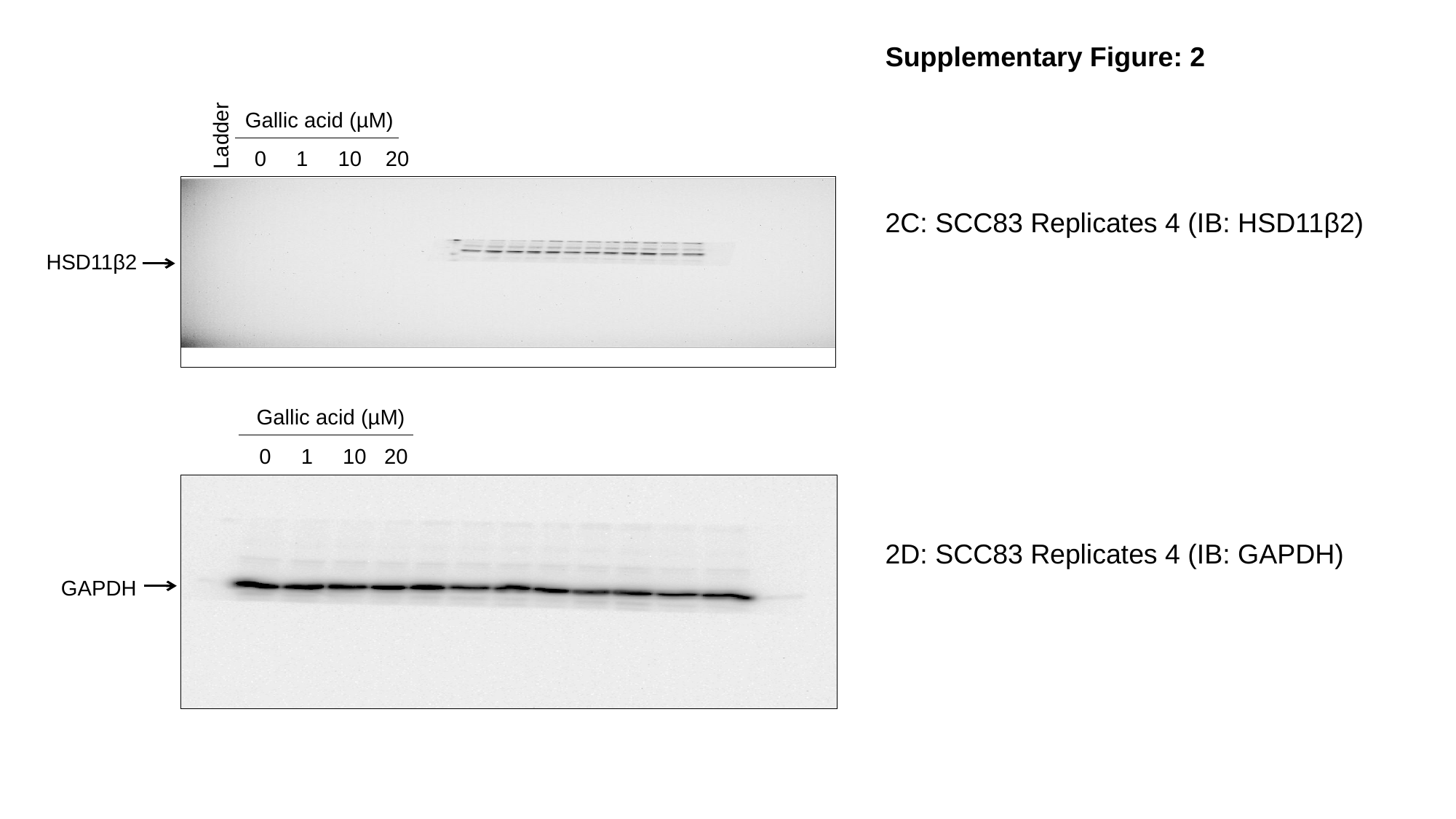

Supplementary Figure: 2
Gallic acid (µM)
 0 1 10 20
HSD11β2
Ladder
2C: SCC83 Replicates 4 (IB: HSD11β2)
Gallic acid (µM)
 0 1 10 20
GAPDH
2D: SCC83 Replicates 4 (IB: GAPDH)
